# Metallographic Characterization of an NBS Spectrometric Low-Alloy Steel Standard

**DOI:** 10.6028/jres.068A.033

**Published:** 1964-08-01

**Authors:** R. E. Michaelis, H. Yakowitz, G. A. Moore

## Abstract

The spectrometric standard steel designated NBS Low-Alloy Steel 461 was investigated by means of electron probe microanalysis and quantitative metallographic techniques employing a digital computer. Electron probe microanalysis showed the steel to be homogeneous in nickel and iron at two to four microns of spatial resolution. The average of all determinations agreed with the certified values for these elements. Inclusions in the steel were identified, classified as to size and shape, and counted. Mean free path data on the inclusions were calculated. The ASTM ferrite grain size number was deduced as 13.5 for the steel in the unetched condition. From the mean free paths in ferrite and pearlite, it was found that the steel is structurally homogeneous at a five micron level. It is concluded that the homogeneity level corresponds closely to the grain size of the material. It is further concluded that NBS-461 steel is sufficiently homogeneous that any present microanalytical technique can be carried out with little chance of inaccuracy duo to inhomogeneity.

## 1. Introduction

Most of the modern rapid instrumental methods of analysis depend on the use of standard samples of composition for calibration. The National Bureau of Standards in cooperation with industrial and government groups, plans, prepares, tests, and certifies selected standards of composition to serve the urgent needs of science and technology in critical areas of calibration.

Whereas generally suitable techniques have been developed for the preparation of homogeneous standard samples of composition to be applied in chemical analysis, or in the usual optical emission and x-ray spectrochemical analysis, [1]^1^ some of the newer analytical methods are far more demanding with respect to the requirement of homogeneity. Included in the newer analytical techniques which have stringent requirements for sample homogeneity at the 1 to 50 *μ* level are those involving the solids mass spectrometer, the laser excitation source in spectrochemical analysis, and the electron-probe microanalyzer. The latter instrument, for example, can perform an analysis with a spot diameter of about 1 *μ.*

An important objective of the spectrochemical standards program is to develop new or improved procedures and methods for preparing, testing, analyzing, and applying the spectrochemical standards. In this, research is directed not only to techniques for providing standards with high measurement excellence, but also to procedures for increasing the applicability of existing and future standards.

The purpose of this report is to present the results of a Limited but critical evaluation of homogeneity at the 2 to 4 *μ* level in one of the available NBS steel spectrochemical standards. The evaluation is intended (1) to provide a more complete characterization of the standard, and (2) to learn of its suitability for application to the newer analytical techniques. The investigation primarily involves a study by the NBS electron probe microanalyzer and by new quantitative metallographic techniques.

## 2. Selection of Material for Study

At present, the NBS does not have available specific standard samples of composition for some of the newer analytical techniques, such as the solids mass spectrometer. However, a number of available NBS standard samples, designed primarily for application in spectrochemical analysis, was prepared and processed to yield material of high homogeneity. Based on the results of extensive testing several years ago by optical emission and x-ray spectrochemical analysis, by chemical analysis, and by metallographic studies, these standard samples were determined to be free from segregation, for the intended applications, for most contained elements. In particular, a set of eight ingot iron and low-alloy steel standards, prepared in the years 1956 and 1957 to contain a graded composition range for about 25 elements, was found to be homogeneous at a level of about 1 mm (1000 *μ*) for most elements as based on the methods of test then available. It was believed that one of these standards would serve the purpose of this investigation.

The study of photomicrographs of these irons and steels indicated structural inhomogeneity for all eight standards at the micron level; however, at least two of the standards appeared to exhibit suitable structural homogeneity at the 10 to 20 *μ* level. The latter level should be satisfactory for application to either the solids mass spectrometer or to the laser excitation source in optical emission analysis.

The material for each standard had been melted in a one-ton induction furnace (high frequency) at the Naval Research Laboratory and cast into a single ingot. In an effort to reduce the number of inclusions, and to improve and standardize the recovery values for the additions, each heat was given a “carbon boil” immediately after melt down. Also, as a possible aid to reducing the inclusions in the final material and to obtaining a finer grain size, a rare earth addition was made to the molten metal in the ladle prior to skimming and pouring into the mold.

Each ingot was processed by forging to a slab having one dimension of the cross section four times that of the other dimension. After cropping top and bottom, 15 and 5 percent respectively, the slab was cut lengthwise and the central longitudinal section corresponding to one-fourth of the slab was discarded. The remaining two slab portions were hot rolled to oversize rods, annealed, straightened, and centerless ground to size. About 900 lb (408.6 kg) of finished rods were obtained for each standard as follows: 100 lb (45.4 kg) of rods 732 in. (5.56 mm) in diameter from the outer section near the bottom of the original ingot; 400 lb (181.6 kg) of rods 1¼ in. (31.75 mm) in diameter from the outer sections near the middle of the original ingot that is currently certified; and additionally, 400 lb of rods 1¼ in. in diameter which will be issued as renewal material when the first 400-lb lots are exhausted.

As would be expected, the rod material 732 in. in diameter received far more severe working than the larger size of 1¼ in. in diameter, hence, the smaller rods exhibited a much finer grain size.

The decision was made to confine the initial investigation by the electron probe microanalyzer and by the new quantitative metallographic techniques to NBS Spectrochemical Standard No. 461. The composition of this steel is shown in table 1.

A random sample was chosen as typical of the entire 100 lb lot. Previous metallographic examination of several selected samples revealed no significant differences.

## 3. Information Desired and Equipment Used

It was desired to determine whether the steel was homogeneous in nickel and iron at micron levels of spatial resolution in the longitudinal, transverse, and normal directions. Furthermore, the average of a set of analyses for these two elements was to be checked against the certified value. For this purpose, the NBS electron probe microanalyzer was employed. This instrument, which enabled analyses to be made at the 2 to 4 *μ* level, has been described in detail elsewhere [2].

Since inclusions were found in the steel, they had to be identified; here both the electron probe microanalyzer and chemical-etching techniques were employed. Additional desirable information concerning the inclusions consisted of their volume percentage in the steel, the mean free path between them, and some idea of their number and size distribution.

Concerning the steel itself, the following information was required: (a) a statement of the apparent ferrite and pearlite percentages in the worked structure of the steel, (b) the mean free path for ferrite and pearlite, and finally (c) the grain size of the steel.

It is apparent that in order to obtain the required information, beyond the mere identification of the inclusions, one must resort to accurate quantitative metallography.

## 4. Experimental Procedure

### 4.1. Electron Probe Microanalysis

To check the chemical homogeneity of the steel, both nickel and iron were investigated for uniformity of composition throughout the sample of 461. With the NBS electron probe microanalyzer, 46 separate spot determinations were made for nickel in the cross section and 62 determinations were made in the longitudinal section. For iron, the corresponding determinations were 9 and 16, respectively. This gives a total of 108 separate nickel determinations and 25 separate iron determinations.

The electron probe microanalyzer was operated at 32.5 keV for all analyses. Specimen currents used were 3.5×10^−8^ (±1%) A for the cross section and 1.0×10^−7^ (±1%) A for the longitudinal section. Observed line to background ratios were 20/1 for pure iron and 30/1 for pure nickel using the second order unresolved *Kα* doublet for these elements in conjunction with an ADP crystal^2^ and a flow proportional counter. Using the first order unresolved *Kα* doublet for nickel in conjunction with a LiF crystal and a G-M counter, the observed line to background ratio was 210/1 for pure Ni. The probe size was 2 to 4 *μ* based on examination of the contamination spots formed.

The x-ray intensities from the sample were compared to those obtained for pure metal end-member standards. The corrections applied were as follows:

Absorption: Philibert [3].
Fluorescence: Birks [4] (enhancement of Fe-*Kα* by 1.73% Ni).
Atomic Number [5]: Not required.
Effect of continuous radiation [6]: Neglected.

An appendix showing the detailed calculations for this paper is available on request [7].

### 4.2. Computer Metallography

A process which makes accurate quantitative metallography economically practical is one which employs a digital computer capable of accepting suitable photomicrographs and of printing out the desired information directly [8]. The computer which has been used to date is the SEAC (Standards Electronic Automatic (Computer). Black and white pictures may be directly introduced in binary machine code by a scanning device. This equipment places a physical size limitation on the photomicrograph to be investigated since the scanning device can accommodate only one picture, 44 mm^2^, at a time.

At present, 28 general operations may be performed on a suitable micrograph. Many of these have been described previously [9]. New operations used in this study are known as “SLICE,” “BLOB TRANSFER,” “BLOB CUSTER,” and “STATISTICS” respectively and these will be described herein.

The photomicrographs to be scanned must have the highest possible contrast between black and white areas because the accuracy of the machine is adversely affected by gray components. The size and shape of the areas to be shown as black must be truly representative of the sample at the magnification chosen. Furthermore, all grain boundaries revealed by etching must be shown as completed networks since the computer will find any openings and count such paired grains as single grains.

The computer scans a raster of square spots 0.25 mm on an edge. For example, if the photomicrograph is taken at a magnification of 500 diam, the computer unit will be 0.25/500 or 5×10^−4^ mm which corresponds to 0.50 *μ*. Therefore, the magnifications used must be known accurately if data such as mean free paths are to be meaningful.

To obtain the metallographic information desired, the machine was required to state the area of the black phase, to display the picture on an oscilloscope screen, to “slice” the picture horizontally and vertically and to perform lineal analyses at right angles to each other. In certain cases, complete “blob” (any discrete black area) analyses were ordered from which complete data on each blob in the picture were deduced and printed out.

To accomplish all of these orders, a master routine is fed into a portion of the computer memory. This will cause the computer to demand a list of orders which are in the form of English words. Given this list, the computer stores the commands and carries them out in the listed sequence. To perform a command, the computer searches the order tape until it locates the matching title. It then reads in the detailed orders and executes this set of orders. The operator, in effect, causes the machine to execute literally thousands of orders in machine language by simply typing in a list of commands in English. To do the complete series of analyses required for obtaining all the information desired about one micrograph of the NBS–461 steel, the simplified English language command list to the computer is:

SCAN. This causes the computer to be supplied with one picture 44 mm^2^. A blank border 1 mm wide is masked onto the picture. The net usable area within the border is 28,224 bits.
AREA. This counts the number of black bits in the image. Division by 28,224 gives the percentage of the phase represented by the image. The area statement is checked by using a calibrated bar chart containing 14,112 black bits which is exactly half the usable picture area of 28,224 bits (704 SEAC words).
DISPLAY. The image which is stored in the computer memory is exhibited on a cathode ray tube.
DUMP. The image in the machine is electrically recorded on wire or tape.
SLICE HORIZONTALLY or SLICE VERTICALLY. The slice operation separately determines the areas of 168 horizontal or vertical line slices. The decimal print out classifies the 168 slices into a histogram of 27 classes. The upper limit of each class and the number of slices found therein is stated.

The area is given in bits and in percent of the net image area (28,224 bits). A *σ* value, based on the histogram of the line slice distribution, is given in percent and the *σ* value of the mean area is given in bits and in percent. The *σ* values may be expected to differ for horizontal and vertical slicings. The larger of the two *σ* values is used.

LINE. This causes the computer to treat the picture as an unbroken horizontal helical raster such as would result from wrapping the picture around a vertical cylinder and joining the right end of each scan line to the left end of the next line. This order produces a decimal table presenting a histogram (on $\sqrt{2}$ classes) of line lengths in the black and white portions of the picture. The mean free path is computed as well as the standard deviations of both path length distributions and of the mean free path.
ROTATE. The picture is turned 90 deg as may be required for a directional interpretation.
LINE. This second LINE order gives the lineal analvsis at right angles to the first LINE order.
BLOB TRANSFER AND RECORD. Each black blob in the picture is analyzed separately. The computer prints out the serial number, area, addresses of the corners of the circumscribed rectangle, height, width, estimated length (*L*), estimated thickness (*T*), and the shape factor (*L/T*).
BLOB CUSTER. This operation reads back the data from the BLOB TRANSFER AND RECORD table together with an image of the single blob and computes the perimeter (*P*) of each blob as well as the complexity factor, *P*^2^/area. The table so constructed contains the input data for the statistical analysis.
STATISTICS. The data from the BLOB CUSTER table are classified into histogram tables of eleven blob parameters on logarithmetically increasing classes of base $\sqrt{2}$. Automatic corrections for blobs cut by the picture edges are made.
END. Machine stops.

### 4.3. Inclusion Counting

The longitudinal and cross sections were polished for inclusion counting, i.e., to reveal the true number, size, and distribution of the inclusions. This was accomplished by performing the final polishing with a quarter-micron diamond powder on micro-cloth for long times at low speeds. The sample was alternately etched and polished and was finished in the unetched condition.

The criteria adopted for the inclusion polishing were as follows: (1) that there be no scratches in the area to be photographed when this area was subjected to rotation in polarized light, (2) that “open circles” were not true inclusions, (3) that the inclusions have sharp boundaries at the magnification of interest. When these criteria were satisfied, it was believed that a true representation of the inclusion concentration had been obtained. After being photographed in this condition, the samples were introduced into the NBS electron probe microanalyzer.

**Figure 1:**
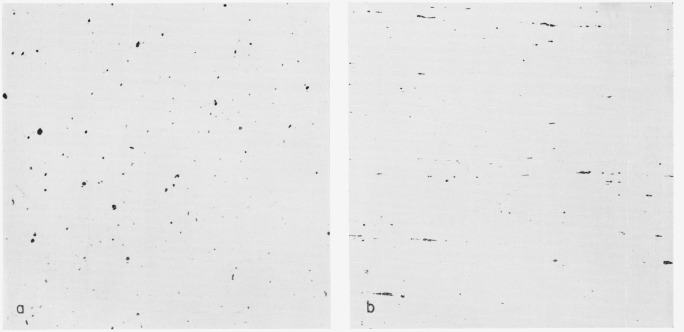
NBS 461 steel polished for inclusion counting and microprobe examination. Cross section, unetched, ×100b. Longitudinal section, unetched, ×100

One criterion for obtaining true photographic records of the surface was that two overlapping areas taken at different exposure times and printed for maximum contrast showed inclusions common to both areas as the same size in both prints. The micrographs used were below the limit where overexposure could conceivably blot out smaller inclusions and reduce the size of larger ones. Furthermore, when sharp black inclusions were obtained against white backgrounds by contact printing, the print truly represented the negative. Finally, the print was visually checked against the sample. When these requirements had been satisfied, the micrographs were ready for computer analysis (figs. 1a and 1b).

Thirteen photomicrographs were processed. Six were of the longitudinal section and seven were of the cross section.

#### Longitudinal section

Photomicrographs were prepared at 50×, 100×, and 200×. The 50× magnification encompassed the width of the rod in two 5×7 photomicrographs. The higher magnifications showed more of the detail of the inclusion shapes, however, the 200× pictures were too high in magnification to give truly representative values. Accordingly, four 50× and two 100× pictures were chosen for examination. Since the computer analyzes a central area on the picture of only 42×42 mm, the pictures were chosen to give maximum, average, and minimum blackness wherever possible. The six pictures chosen were:

50×−maximum blackness 50×−minimum blackness
50×−average blackness 100×−maximum blackness
50×−average blackness 100×−minimum blackness

The computer was ordered to furnish the area of the inclusions, to display them on the oscilloscope, to slice the pictures horizontally and vertically, and to perform two lineal analyses of the pictures at right angles to each other.

#### Cross section

The area, display, slice, and lineal analyses were performed on seven pictures, six of which were at 100× and one at 50×. The micrographs were taken such that the cross section was covered from one edge to nearly the other edge. The six 100× pictures represented three from the center and three from the edge. These were taken for maximum, average, and minimum blackness from each position. The 50× picture was randomly selected near the middle.

### 4.4. Studies on Etched Structures

Super picral, nital, and high-chlorine aqua regia failed to cut cleanly through all of the grain boundaries; however, a 3½ percent solution of concentrated HNO_3_ in water successfully performed this task. Etching time was 9 to 10 s with the sample immersed in the solution.

In addition to normal magnifications, the specimens were photographed, in some cases, at magnifications such that the statistics table printed out by the computer would classify the grain sizes directly into ASTM numbers. Since the computer program establishes $\sqrt{2}$ size classes on the basis of *maximum* blob area, and ASTM Method E-112 [10] defines grain size values in terms of *average* grain areas, an adjustment by $\sqrt[{4}]{2}$ on diameters or $\sqrt[{8}]{2}$ on areas causes the two classifications to match. The adjustment factor was found to be nearly constant for each of the 30 listed ASTM classes. This factor is 0.925, i.e., if one were doing a grain size count at 1000× by conventional methods, a photomicrograph taken at 925× and processed by the computer would give the same grain size. Based on these facts, photomicrographs were taken of both the longitudinal and cross sections at 462.5×, 500×, 925×, and 1000×. To increase the contrast, the 8 × 10 photographs were printed onto lithographic film and then printed onto high contrast photographic paper. This revealed the pearlite as white and the ferrite as black. Photomicrographs at 500× are shown in figures 3 and 4. Computer analyses were made on portions of each photomicrograph except those of the longitudinal section taken at 925× and 1000×.

For these analyses, the computer was ordered to state the area of ferrite (black), display the picture, slice horizontally and vertically, and to do lineal analyses at right angles to one another. On a photomicrograph of the cross section taken at 462.5×, the computer was ordered to do a full blob analysis and report the results in the “Statistics” table. The information to be gained by these procedures is the ferrite percentage, the mean free path in microns for ferrite and pearlite, and the grain size. Furthermore, from the mean free path data and from the manner in which the photomicrographs were prepared, data for a grain size determination by the Heyn intercept method are available [10].

## 5. Results

### 5.1. Homogeneity

#### a. Nickel in the Cross Section

Microprobe analyses were made on three separate days. First, 15 random points on the sample were analyzed, and then two traces across the sample were made at right angles to one another on successive days. These traces contained 19 and 12 points respectively. Points taken just inside the edge of the sample gave results which were very different from results taken from points a millimeter or more away from the edge. There were nine such points, of which eight gave high results and one gave a low result. The latter probably can be explained by the fact that the beam overlapped into the mounting material. The edge was not maintained completely flat during polishing. This resulted in a higher x-ray takeoff angle and caused the high results observed for the remaining eight points.

Due to the low nickel concentration and the low x-ray emergence angle in the NBS microanalyzer (5.5°), the 3*σ* counting error was about 0.45 wt percent. Table 2 shows the expected concentration ranges and the observed ranges after the edge affected points have been dropped.

#### b. Iron in the Cross Section

The observed range for the iron concentrations was 95.6 to 98.2 percent with an average of 97.0 percent. The iron concentration, by difference, based on the certificate is 96.5 percent. The largest deviation from the observed mean value is 1.44 percent. The appendix [7] contains the complete data.

#### c. Nickel in the Longitudinal Section

Two separate investigations were made; these consisted of 48 and 14 analyses respectively. Of the total of 62 points, 60 fell within the expected limits. The average concentration found for both investigations was 1.73 percent Ni, which agrees with the certified value. The extreme edges were avoided. The complete data for the 62 random points investigated are shown in the appendix [7], while table 3 shows the expected and observed concentration ranges.

#### d. Iron in the Longitudinal Section

Sixteen analyses were made at random points with the extreme edges being avoided. The observed concentration range was 93.0 to 98.6 percent with an average of 95.8 percent. Two points deviated from the mean by 2.9 percent; the next largest deviation was 1.6 percent. The appendix [7] contains the complete data.

### 5.2. Inclusions in the Steel

#### a. Identification of the Inclusions

The longitudinal section of the steel shows stringers which appear to be composites under the microscope. The structure appears somewhat similar to that observed in eutectoid type structures, i.e., barring occurs. These inclusions were investigated by means of the electron probe with the result that Mn and Cr were confirmed in them. There is no Ni present in the inclusions. There appeared to be Fe, but this is indeterminate because the matrix is so rich in Fe and the distribution in depth of the inclusions is unknown.

**Figure 2:**
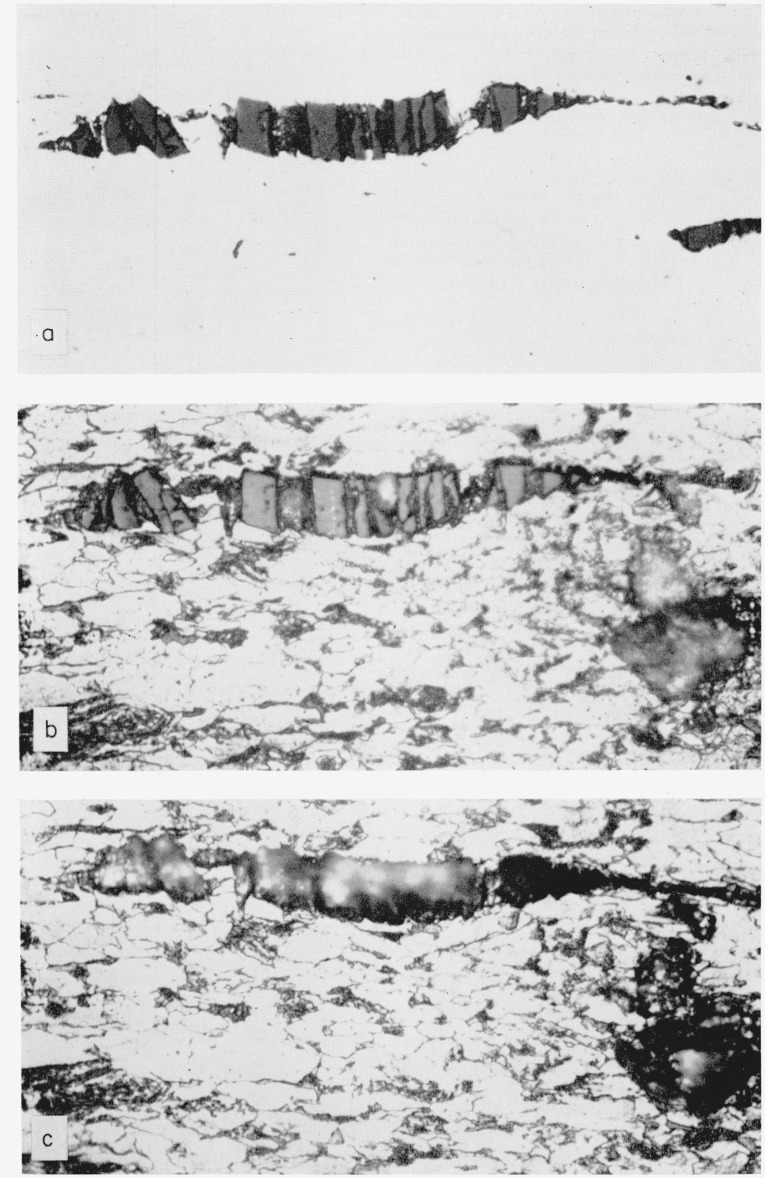
Typical inclusion in longitudinal section. Unetched, ×1000. Note the definite duplexity, i.e., discrete bars with smaller crystallities betweenSame inclusion after succesive etching for 10 s in 10 percent nital, 5 min in 10 percent aqueous chromic acid (neither of which attacked the inclusions), followed by 5 min in boiling alkaline sodium picrate, and 5 min in 5 percent alcoholic hydrochloric acid. Note attack by the sodium picrate on the small crystallites between bars and slight attack (lighter color) on bars by the hydrochloric acid, indicating presence of FeO·MnO largely in the crystallites. ×1000.Same as (b) except followed by 10 min in 20 percent aqueous hydrofluoric acid. Note nearly complete removal of bars indicating presence of FeO·SiO_2_. ×1000.

An attempt was made to further identify the inclusions by progressive etching [11]. This scheme was followed until nearly all of the inclusion components had been removed or attacked, i.e., through a 10 min etch in aqueous 20 percent HF. Photomicrographs were taken of the same inclusion at each stage. Some of these are shown in figure 2. From the results it was concluded that: (1) The bars are largely FeO·SiO_2_, but they probably contain some MnO·SiO_2_ since the bars are slightly attacked by 5 min in 5 percent HCl in C_2_H_5_OH. (2) The other components are probably MnO·FeO (note attack by 5 min in boiling alkaline sodium picrate) mixed with a carbide. It is surmised that the carbide is Fe-Cr since the carbon bearing patches in the bulk of the steel behave in a like manner under polarized light to the crystallites remaining after the FeO·SiO_2_ bars have been removed by 20 percent HF. It may be that these are only an iron carbide and that the Cr is combined as oxide or carbide.

#### b. Volume and Weight Percentages of Nonmetallic Inclusions

##### Longitudinal Section

The area percent of a random cut has been shown to be equal to the volume percent of the material [12]. Considering the previously established homogeneity of NBS–461 steel, it was believed that virtually any cut made on the random sample would be truly representative of the standard. Area results as given by the computer are then interpreted to be volume percent of inclusions in the steel. The average of four 50× pictures gave a volume percentage of inclusions equal to 0.40. A single 100× picture chosen for average blackness (inclusion density) gave a volume percentage of 0.42.

##### Cross Section

Two sets of photomicrographs were taken near the center and near the edge. These were processed by the computer as separate entities to determine whether any large gradients of inclusion density existed. For the set of three 100× pictures taken near the center of the sample, the volume percentage of inclusions was 0.38 percent. The three 100× pictures taken near the edge gave an average volume percentage of 0.42 percent. Obviously, no appreciable gradient was found, and the six pictures were then taken as representative of the entire cross section. This gave an average volume percentage of 0.40 percent. Note that this is the same result as that obtained for the longitudinal section. Data for each picture processed will be found in the Appendix [7].

#### c. Estimation of the Weight Percent of Inclusions

From the results, on both the longitudinal and cross sections it is reasonable to say that the inclusion content is 0.40 volume percent. For an approximate calculation of the weight percentage of the inclusions, equal portions of FeO·SiO_2_ and FeO·MnO can be assumed in the inclusion. The density of FeO·SiO_2_ is 3.5 while that of FeO·MnO is 5.6. The average density is then about 4.5. The density of the metallic portion of the steel is about 7.9. Then for the total density of the steel as issued:

*p* = [(0.996) (7.9) + (0.004) (4.5)]
*p* = 7.8384+0.0180
*p* = 7.8564

Weight percent of inclusions of 50 percent FeO·SiO_2_ and 50 percent FeO·MnO is 0.0180/7.8564=0.23 percent. Thus, 0.23 weight percent of inclusion is obtained. To obtain a better indication of the absolute range for the weight percentage of the inclusions, the value was computed by first assuming the inclusions to be only FeO·SiO_2_ and then to be only FeO·MnO. The weight percent in the first case is 0.18 and in the second, 0.28. It can thus be stated with confidence that the weight percentage of the inclusions is 0.23±0.05.

#### d. Estimation of Number and Size of Inclusions

The computer was ordered to do a full statistical analysis on the average 100× picture taken of the cross section near the center. From this analysis, some estimation of the number and size of the inclusions can be made.

The statistics table indicated that the average height and width of the inclusions was equal and had a value of 5.25 *μ.* Furthermore, the mean value of the major axis of elliptical shapes was 6.0 *μ*. Based on this, it is possible to view the inclusions in the cross section as sections of an elliptical cylinder having a mean diameter of approximately 5.5 *μ.*

Data on the area distribution of the inclusions from the statistical analyses, coupled with the fact that at 100× the real area of metal represented by the photomicrograph is 0.176 mm^2^, enabled the calculation of the approximate number of inclusions per cubic millimeter of steel. This was first attempted by using the method of Bergh and Lindberg [13] which assumes that the inclusions are spherical. However, the summation of the predicted spherical volume of the inclusions was over 100 percent greater than the real volume as deduced by the computer. Therefore, the Bergh and Lindberg method was discarded.

Visual observation indicates that the inclusions in NBS–461 steel are cylindrical in shape. On this basis, an attempt was made to find the average length in terms of the diameter. Both mean free path data in the inclusions, and the statistical analyses of longitudinal and cross-sectional photomicrographs were used. The results from the former gave the length equal to 2.64 times the diameter, while the results from the latter gave the length as 2.73 times the diameter. For purposes of calculation, the length was taken as 2.7 times the diameter. The conversion of the number of inclusions in a given class per square millimeter to inclusions of the same class per cubic millimeter is then obtained by dividing by the particle length in millimeters. Since, for a 100× photomicrograph, the computer scans spots 2.5 *μ*^2^, the smallest observable inclusion diameter is 2.5 *μ*, as compared with 10 *μ* minimum diameter frequently reported.

The total number of inclusions per cubic millimeter was found to be about 14,000 of which 76 percent had diameters of 5 *μ* or less. These 76 percent represent only about 20 percent of the total inclusion volume, however. The total inclusion volume percent was 0.44 from the photomicrograph in question; the calculated inclusion percentage was also 0.44.

A table of probabilities for a 50 *μ* spherical volume to contain one inclusion of each size observed was prepared. The 50 *μ* spherical volume was chosen to be representative of the sample size for laser excitation sources and the solids mass spectrometer. Full data are shown in the Appendix [7].

#### e. Mean Free Path Between Inclusions

The computer delivered data on the mean free paths in both the inclusions and in the steel, as obtained from lineal analyses of the photomicrographs. In the cross section, the average mean free path within an inclusion was about 4.5 *μ*, while the average mean free path in the steel was about 1600 *μ*. From these figures the probability of a random 1 *μ* electron probe touching any inclusion is only about one in 350.

In the longitudinal section, the average mean free path in an inclusion was about 7 *μ* while in the steel, it was about 2700 *μ*. Here the probability for a random 1 *μ* electron probe to touch an inclusion is only about one in 380. The fact that the mean free paths are smaller than the maximum dimensions of the inclusions is to be expected since the longest path through the inclusion will not always be the path chosen by the computer. Complete mean free path data for each picture processed will be found in the Appendix [7].

### 5.3. Analysis of the Etched Structure in NBS-461 Steel

#### a. Ferrite and Pearlite Percentages

It is readily apparent from the photomicrographs of the etched structure that this steel is extremely fine grained. This presents an immediate dilemma since a fairly large portion of the grain itself may be lost in etching to clean cut grain boundaries. Thus, the apparent ferrite percentage derived from a photomicrograph of the etched structure can be very much lower than the actual ferrite percentage. As an illustration for a steel with an ASTM grain size number of 6.0, the average grain diameter is given as 45 *μ* [10]. Etching a 1 *μ* grain boundary into such a steel causes the loss of 145 (½ *μ* on each “side”) or 2.2 percent of the grain diameter and 4.4 percent of the area. However, in a steel with ASTM grain size of say 13, the average grain diameter is about 4 *μ* and etching a 1 *μ* boundary into such a steel causes the loss of 25 percent of the grain diameter. The resulting area loss is about 44 percent.

The problem, then was to obtain the ferrite and pearlite percentages with and *without* the grain boundaries. Fortunately, this was possible with the computer display system. Each picture as scanned into the computer was displayed on a cathode ray tube and compared with the original. The sensitivity of the scanner was adjusted until all of the grain boundaries were clearly cut through; the area deduced by the computer under these conditions was the ferrite percentage in the etched condition Then the pictures were redisplayed and the scanner sensitivity adjusted until the grain boundaries were apparently removed (this was when the calibrated bar chart gave an area value of nearly 14,112 bits). No particular encroachment on the pearlite areas could be discerned by visual comparison of the cathode ray tube and the actual photomicrograph. The area deduced by the computer under these conditions was considered to be the ferrite percentage in the “unetched” condition, i.e., the actual ferrite percentage in the steel.

In the etched condition, the average volume percent of ferrite was found to be 32.8 percent, while in the unetched condition, the average volume percent of ferrite was found to be 54.8 percent. From this, two things are apparent: etching this fine grained steel has reduced the apparent ferrite surfaces by more than 40 percent. Furthermore, the pearlite percentage seems very high for a steel containing 0.15 percent carbon. This high pearlite value is a result of the history of the steel especially the fact that it is not fully annealed. For an estimate of the pearlite percentage which would be apparent in a photomicrograph of the annealed structure, NBS–461 steel may be compared to an 8615 steel which has roughly the same composition. In 8615 steel, the eutectoid value is at approximately 0.65 percent carbon. From this, an approximate value of 23 percent pearlite would be expected. Thus, as a result of its history, the NBS-461 steel exhibits approximately twice as much pearlite as would be anticipated in the fully annealed structure. Complete data on ferrite percentages in all pictures processed will be found in the Appendix [7].

#### b. Mean Free Paths for Ferrite and Pearlite

The computer was ordered to do lineal analyses on the steel in the etched condition. The average ferrite mean free path was found to be 1.63 *μ* while the average pearlite mean free path was found to be 3.38 *μ.* From these data the probabilities for a 1 *μ* electron probe to be in a given constituent can be computed. The probabilities are about one in five that the spot is in pure ferrite, one in five that it is in a ferrite-pearlite mixture, and three in five that it is in pure pearlite. With a 3.5 *μ* electron probe, one is always in a mixture of ferrite and pearlite. Complete mean free path data on all pictures processed will be found in the Appendix [7].

#### c. Grain Size of the Steel

Despite the fact that the steel was in a worked condition, it was felt that a meaningful grain size could be deduced. Furthermore, with the aid of the computer, a number of individual grains could be analyzed separately and a histogram of ASTM grain classes in the steel could be established.

It was desired to state the grain size in both the etched and unetched conditions; the grain size deduced for the unetched condition is believed to be the more accurate statement for the real grain size. However, since one normally works with etched structures when using the ASTM grain size chart, it was deemed necessary to present both values. With both values available some idea of the effect of etching on the apparent ASTM grain size in a very fine grained steel could be indicated.

To obtain the grain size in the etched condition, the Heyn intercept procedure was adopted. The average intercept lengths required are the same as the mean free paths given by the computer. From these data, the number of grains per cubic millimeter may be calculated by the following relation [10].

$$n_{1\;tn}=\left(0.7\right)\;\left(n_{1}\right)\;\left(n_{t}\right)\;\left(n_{n}\right)$$

where

*n*_1_*_tn_*: is the number of grains per cubic millimeter.
*n*_1_: is the average number of grains per millimeter intercepted in the longitudinal direction.
*n_t_*: is the average number of grains per millimeter intercepted in the transverse direction.
*n_n_*: is the average number of grains per millimeter intercepted in the normal direction.

**Figure 3:**
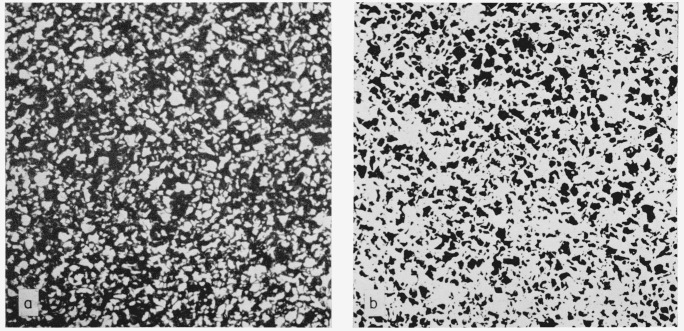
Cross section of NBS 461 steel etched for 9 s in 3-1/2 percent *HNO_3_* Ferrite white and pearlite black. ×500Same as (a) except ferrite black and pearlite white as scanned into the computer. ×500

**Figure 4:**
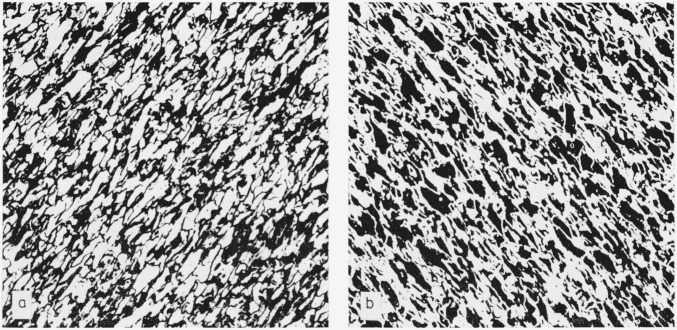
Longitudinal section of NBS 461 steel etched for 10 s in 3-1/2 percent *HNO_3_* Ferrite white and pearlite black. ×500Sameas (a) except ferrite black and pearlite white as scanned into the computer. ×500

Since photomicrographs had been prepared from sections of the steel taken at right angles to one another and since rotations had been performed in the computer, all of the necessary data were available. The values for *n*_1_ and *n_t_* were taken from the ferrite mean free path data in the longitudinal section while the *n_n_* value was taken from the ferrite mean free path data in the cross section. Duplicate grain size analyses were made on photomicrographs taken at 462.5× and 500×. The latter are shown in figures 3 and 4. The calculation made on the 462.5× picture will serve to illustrate.

$$n_{1\;tn}=\left(0.7\right)\;\left(n_{1}\right)\;\left(n_{t}\right)\;\left(n_{n}\right)$$

Since 1 mm equals 1000 *μ*, the *n* value is in general equal to:

$$n=\frac{1000}{\text{mean free path}\;\left(\text{in microns}\right)\;\text{for ferrite}}$$

In the 462.5× photomicrograph the mean free path values were 2.03 *μ* in the longitudinal direction, 1.48 *μ* in the transverse direction, and 1.98 *μ* in the normal direction. Thus

$$\begin{array}{l} n_{1\;tn}=\left(0.7\right)\;\left(\frac{1000}{2.03}\right)\left(\frac{1000}{1.48}\right)\left(\frac{1000}{1.98}\right)\\ n_{1\;tn}=117\times 10^{6}\;\text{grains}/{\text{mm}}^{3}. \end{array}$$

Since ASTM grain size 14 has 45.2×10^6^ grains/mm^3^ and grain size 15 has 128×10^6^ grains/mm^3^, it can be seen that the grain size in the etched condition is indicated to be 14.9. Precisely the same result is obtained when the mean free path data from the 500× photomicrographs are used.

To obtain the grain size in the unetched condition, mean free path data for the ferrite in the unetched condition were required. These data were obtained by using the ferrite area values obtained in the unetched condition in combination with the total number of lines found in the ferrite by the lineal analysis performed with the computer on the etched structure. The effect is to add the grain boundary back onto each grain. In our example for the 462.5× picture, the new mean free path values found were: 3.29 *μ* in the longitudinal direction, 2.55 *μ* in the transverse direction, and 3.65 *μ* in the normal direction. These values led to an *n*_1_*_tn_* value of 22.9×10^6^ grains per cubic millimeter corresponding to an ASTM grain size of 13.3. When adjusted mean free path data were used in conjunction with the 500× photomicrographs, the ASTM grain size for the unetched condition was found to be 13.5.

Virtually the same effect, i.e., the addition of the boundary area to the grain area, can be obtained by adding the perimeter of the etched grain to the area of the etched grain. To obtain an indication of the spread of grain sizes in this worked material, the computer was ordered to do a full statistical analysis, which includes the perimeter *plus* area data, on a photomicrograph of the cross section taken at 462.5×. In this case it was hoped that all the grain boundaries were cleanly cut through since any connectivity will be found by the computer and treated as one unit. Of a total of 356 blobs analyzed by the computer, only about 30 exhibited this type of complexity as evidenced by a second peak in the histogram of frequency versus ASTM grain size.

**Figure 5:**
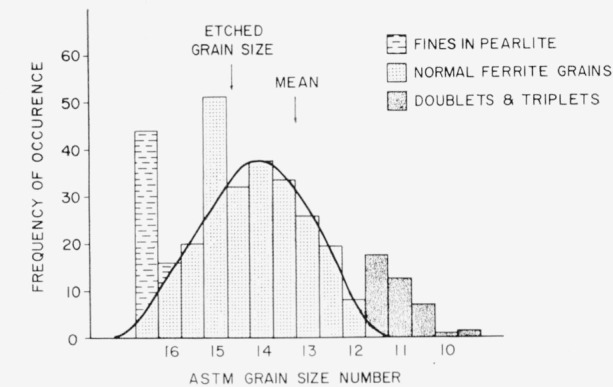
Histogram of ASTM grain size numbers in NBS 461 steel. The etched grain size is 14.9 and the mean unetched grain size is 13.5.

The histogram is presented in figure 5. The peaks associated with the very small grain sizes are ferrite particles in the pearlite while the larger grain sizes are due to the previously mentioned complex grains. The estimated Gaussian distribution curve is also shown. From this figure, the mean ASTM grain size is 13.2.

Thus, in the etched condition, the apparent rounded ASTM grain size is 15 while in the unetched condition, the average grain size is 13.2 to 13.5. The significant size range indicated by the Gaussian distribution is about ASTM size 12.5 to ASTM size 15.5. It is interesting to note that there are about two thousand grains for each inclusion.

## 6. Discussion

It has been found that NBS-461 steel is homogeneous in both nickel and iron at 2 to 4 *μ* levels by means of electron probe microanalysis. These results have been corroborated by Adler^3^ who made 10 determinations each for nickel and iron in the cross section of the steel using a, 1 *μ* electron probe. The maximum volume of steel excited was assumed to be about 7 *μ*^3^ (a hemisphere 3 *μ* in diameter). This corresponds to about 5×10^−11^ g of steel per determination. The average of a number of determinations for nickel and iron in both the cross section and the longitudinal section was in close agreement with the certified value of 1.73 percent for nickel and with the predicted value for iron (by difference) of 96.5 percent.

The operator of an electron probe microanalyzer can easily avoid visible inclusions; even if he could not, the probability for striking an inclusion with a 1 *μ* probe is only one in 350. Furthermore, if the x-ray path from the sample is assumed to pass through a 20 *μ*^3^ hemisphere of steel before the x rays emerge from the surface, the probability for an underlying inclusion to be anywhere in the x-ray path is less than 3 in 100.

The steel has been shown to be structurally homogeneous at a 5 *μ* level since the mean free path in ferrite is 1.63 *μ* while the mean free path in pearlite is 3.38 *μ.* Thus, any 5 *μ* probe will be in a structurally homogeneous region. Furthermore, other metallographic observations have shown that there is no long range inhomogeneity in the structures. On this basis it is felt that at a 5 *μ* level, the structural homogeneity predicts that the other elements in NBS-461 steel, except those concentrated in the inclusions, will be uniformly distributed.

It is reasonable to expect partition of some elements between ferrite and carbide (or in pearlite). For example, manganese, phosphorus, sulfur, and silicon are known to concentrate in ferrite areas while chromium, vanadium, niobium, tungsten, and tantalum are known to have an affinity for carbide regions. Therefore, at less than the 5 *μ* structural homogeneity level, these elements might well give fluctuations in analysis.

For other microanalytical techniques, a 50 *μ* sphere was assumed to be a representative sample. This corresponds to about 0.52 microgram of the steel or about ten thousand times the amount investigated by means of electron probe microanalysis. Since it takes about four inclusions of average size to make a 1 percent discrepancy in weight, such an occurrence is highly unlikely.

A commonly found grain size in many steels corresponds to ASTM number 5.5 or 6.0. In this case, the 50*μ* spherical volume would sample only one or two grains. However, it has been shown that the average grain size of NBS-461 steel corresponds to ASTM number 13.5. The resultant average number of grains in the 50*μ* sphere is about three thousand—certainly a fair sampling for an analysis.

It is believed that the homogeneity level of this steel corresponds very closely to the grain size. In this case, the fine grain size was achieved by the addition of rare earths, aluminum, and other beneficial elements. Extensive cold-working was followed by a final process anneal just sufficient to produce recrystallization without grain growth.

It can be confidently stated that the homogeneity level in NBS-461 steel is such that present day microanalytical techniques can be carried out with little fear of discrepancies due to inhomogeneity.

## 7. Summary of Results on NBS-461 Steel

### 7.1. Homogeneity

#### Nickel

108 separate Ni determinations were made with the microprobe (2–4 *μ* spot). Of these only two fell outside of 3*σ* limits predicted by the number of counts. The longitudinal, transverse and normal directions were examined. The average analysis was 1.73 percent Ni which agrees with the certificate value.

#### Iron

25 separate iron determinations were made. Again all three directions were examined. The largest deviation from the mean was 2.9 percent. The average analyses were 95.8 percent iron in the longitudinal section and 97.0 percent iron in the cross section. The certificate value (by difference) is 96.5 percent Fe.

#### Other Elements

Probably homogeneous at 5 *μ* levels except for those contained in the inclusions.

### 7.2. Inclusions

Identification: Both the microprobe and chemical etching tests were used. The inclusions were identified as consisting of FeO·SiO_2_ and FeO·MnO. Some Cr is also present in a combined form.

Volume percentage: 0.40

Weight percentage: 0.23 ±0.05 (assumed equal portions of FeO·SiO_2_ and FeO·MnO on a weight basis).

Mean free path in inclusion: 4.7*μ* (cross section); 7 *μ* (longitudinal section); 5.75*μ* average.

Mean free path in steel: 1600*μ* (cross section); 2700*μ* (longitudinal section); 2100*μ* average.

Number of inclusions per cubic millimeter: 14,000 (approx.).

Probability for random one micron probe to strike a surface inclusion: 1 in 350.

Probability for x rays from a microprobe to strike an underlying inclusion: 1 in 34.

Probability for an inclusion to affect an analysis of a 50*μ* sphere of steel by 1 percent: Negligible.

Inclusion shape: Rod with average length equal to 2.7 times the average diameter.

### 7.3. Characteristics of the Steel

Ferrite percentage: 54.8

Apparent ferrite percentage after etching: 32.8

Mean free ferrite path: 1.63*μ*

Mean free pearlite path: 3.38*μ* (thus a 3.5*μ* spot must always be in a mixture; a 1*μ* spot has a one in five probability of being in pure ferrite and a three in five probability of being in pearlite).

Average ASTM grain size in etched condition: 14.9 (by Heyn method).

Average ASTM grain size in unetched condition: 13.5 (Heyn and particle area methods).

Average ferrite grain diameter (unetched): 3.5*μ*

## Figures and Tables

**Table 1:** NBS Spectrographic low-alloy steel standard No. 461 Provisional certified analysis (November 25, 1959)

Element	Wt
	
	%
Fe	*(96.47)
C	0.15
Mn	.36
P	.053
S	(.02)
Si	.047
Cu	.34
Ni	1.73
Cr	0.13
V	.024
Mo	.30
Sn	.022
Ti	(.01)
B	0.000_2_
As	.028
W	.012
Zr	(<.005)
Nb	.011
Ta	.002
Al	(.005)
Co	.26
Pb	(.003)
Ag	(.001_5_)
Ge	(.001_5_)
O	(.02_0_)
N	(.00_6_)

* Values in parenthesis are *not* certified, but are given for information on the composition. Iron percent is by difference.

**Table 2:** Nickel concentration in cross sections of NBS-461 steel*

Run number	Number of points	Expected limits**	Observed limits	Average concentration
				
		%	%	%
1	12	1.47–2.35	1.50–2.38	1.85
2	15	1.30–2.19	1.33–2.12	1.74
3	10	1.28–2.22	1.48–2.15	1.75

* See Appendix [7] for complete data.

** Range shown is the certified value (1.73%) ±3 times the standard counting error.

**Table 3:** Nickel concentration in longitudinal sections of NBS-461 steel

Run number	Number of points	Expected limits	Observed* limits	Average concentration
				
		%	%	%
1	48	1.22–2.23	1.19–2.05	1.73
2	14	1.35–2.12	1.27–2.03	1.73

* One point in each run was too low.

## Footnotes

1 Figures in brackets indicate the literature references at the end of this paper.

2 ADP refers to an ammonium dihydrogen phosphate crystal, which has a 2d spacing of 10.65 Å.

3 I. Adler, private communication (1963).
